# Additive effects of a small molecular PCNA inhibitor PCNA-I1S and DNA damaging agents on growth inhibition and DNA damage in prostate and lung cancer cells

**DOI:** 10.1371/journal.pone.0223894

**Published:** 2019-10-10

**Authors:** Shan Lu, Zhongyun Dong

**Affiliations:** Division of Hematology-Oncology, Department of Internal Medicine, University of Cincinnati College of Medicine, Cincinnati, Ohio, United States of America; University of South Alabama Mitchell Cancer Institute, UNITED STATES

## Abstract

Proliferating cell nuclear antigen (PCNA) is essential for DNA replication and repair, and cell growth and survival. Previously, we identified a novel class of small molecules that bind directly to PCNA, stabilize PCNA trimer structure, reduce chromatin-associated PCNA, selectively inhibit tumor cell growth, and induce apoptosis. The purpose of this study was to investigate the combinatorial effects of lead compound PCNA-I1S with DNA damaging agents on cell growth, DNA damage, and DNA repair in four lines of human prostate and lung cancer cells. The DNA damage agents used in the study include ionizing radiation source cesium-137 (Cs-137), chemotherapy drug cisplatin (cisPt), ultraviolet-C (UV-C), and oxidative compound H_2_O_2_. DNA damage was assessed using immunofluorescent staining of γH2AX and the Comet assay. The homologous recombination repair (HRR) was determined using a plasmid-based HRR reporter assay and the nucleotide excision repair (NER) was indirectly examined by the removal of UV-induced cyclobutane pyrimidine dimers (CPD). We found that PCNA-I1S inhibited cell growth in a dose-dependent manner and significantly enhanced the cell growth inhibition induced by pretreatment with DNA damaging agents Cs-137 irradiation, UV-C, and cisPt. However, the additive growth inhibitory effects were not observed in cells pre-treated with PCNA-I1S, followed by treatment with cisPt. H_2_O_2_ enhanced the level of chromatin-bound PCNA in quiescent cells, which was attenuated by PCNA-I1S. DNA damage was induced in cells treated with either PCNA-I1S or cisPt alone and was significantly elevated in cells exposed to the combination of PCNA-I1S and cisPt. Finally, PCNA-I1S attenuated repair of DNA double strand breaks (DSBs) by HRR and the removal of CPD by NER. These data suggest that targeting PCNA with PCNA-I1S may provide a novel approach for enhancing the efficacy of chemotherapy and radiation therapy in treatment of human prostate and lung cancer.

## Introduction

Proliferating cell nuclear antigen (PCNA) is an evolutionally very well conserved multifunctional protein [1, 2] and a non-oncogenic protein essential for tumor cell growth and survival. It is overexpressed in all tumors [2]. Overexpression of PCNA in prostate cancer [3, 4] and non-small cell lung carcinoma (NSCLC) [5] is associated with advanced disease and metastasis, and is a reliable biomarker predicting poor prognosis of cancers of various tissue types [3, 4, 6–8]. Given that tumor cells are more active in replication and contain much higher levels of damaged DNA [9, 10] than normal cells, they are more vulnerable to the stress of downregulation or inhibition of PCNA function. Therefore, targeting PCNA could be an effective approach for treatment of cancer.

Native PCNA, present predominantly in the nucleoplasm as “free-form PCNA”, is a ring-shaped homotrimeric protein joined together through head to tail interaction [11, 12]. To be functional, PCNA must be linearized or monomerized, and relocalized. Upon being loaded onto the primer-template junctions of DNA, PCNA encircles DNA, serves as a platform for and interacts with proteins involved in DNA replication and repair and other cellular processes [2, 13–16]. When monomerized and exported to cytoplasm, PCNA was shown to interact with procaspases to inhibit apoptosis [17] and with glycolytic enzymes to promote glycolysis [18]. PCNA also interacts with some cell signaling proteins, such as PI3K proteins, and regulates cell signaling processes [19]. On cell membrane, PCNA interrupts the recognition of tumor cells by natural killer cells [20]. PCNA interacts with its partner proteins containing PIP (PCNA interaction protein)-box, KA-box, APIM (AlkB homologue 2 PCNA-interacting motif), and other motifs [2, 16, 19].

Great efforts have been made to develop novel approaches targeting PCNA for cancer therapy. Peptides mimicking the APIM or a sequence of caPCNA (“cancer associated PCNA”) selectively inhibit tumor cell growth, induce apoptosis, and enhance cytotoxicity of chemotherapy drugs on tumor cells [19, 21–23]. The selective inhibitory effects were also observed in cancer cells treated with small molecule T2AA targeting the PIP-box [24, 25] and small molecule AOH1160 targeting caPCNA [26]. Targeting PCNA in replisomes with monoclonal antibodies triggers lethal DNA replication stress in tumor cells [27]. The PCNA-targeting peptides and small molecule (AOH1160) are well tolerated in animals and show the therapeutic effects against various types of tumors, especially when combined with DNA damage drugs [19, 21, 23, 26, 28].

Targeting PCNA with peptides and small molecules on a single motif described above only interrupts PCNA interactions with certain partner proteins and, hence, compromises some functions of PCNA. Given that PCNA must be relocalized to execute its functions, we hypothesized that targeting PCNA relocalization could be a more effective approach. We have identified a novel class of small molecules (termed PCNA inhibitors, PCNA-Is) that bind directly to PCNA trimers at the interfaces of two monomers, stabilize the trimer structure, and interfere with PCNA relocalization [29]. The two lead compounds PCNA-I1 and PCNA-I1S selectively inhibit tumor cell growth and induce apoptosis in tumor cells at nanomolar concentrations. Tumor cells of various tissue types, including cells with p53 deletion or mutation and cells with multidrug resistant phenotype, are all susceptible to the PCNA inhibitors [29–31]. Moreover, PCNA-I1 therapy significantly retards the growth of human prostate cancer xenografts in mice and does not cause apparent toxicity to the hosts [31]. Induction of DNA damage by PCNA-I1 and PCNA-I1S reveals the underlying molecular mechanism by which the compounds inhibit cell growth. The purpose of this study was to investigate the combinatorial effects of PCNA-I1S with DNA damaging agents on cell growth, DNA damage, and DNA repair. We found that treatment of PCNA-I1S in combination with DNA damaging agents Cs-137, UV-C, or cisplatin (cisPt) produced additive inhibitory effects on growth of prostate cancer and NSCLC cells. PCNA-I1S attenuated PCNA association to chromatin stimulated by H_2_O_2_ in quiescent cells, induced DNA damage, promoted DNA damage induced by cisPt, and inhibited DNA repair mediated by the homologous recombination repair (HRR) and the nucleotide excision repair (NER). Thus, targeting PCNA with PCNA-I1S may provide a novel approach for enhancing the efficacy of DNA damaging chemotherapy and radiation therapy in treatment of cancer.

## Materials and methods

### Reagents

MTT [3-(4,5-dimethylthiazol-2-yl)-2,5-diphenyltetrazolium bromide], H_2_O_2_, and cisPt were purchased from Sigma Aldrich (St. Louis, MO). Antibodies against PCNA, α-tubulin, histone H1, and phospho-histone H2AX (Ser139) named as γH2AX were purchased from Cell Signaling Technologies (Danvers, MA). OxiSelect^™^ Cellular UV-Induced DNA Damage Staining kit and OxiSelect^™^ Comet assay kit were purchased from Cell Biolabs, Inc (San Diego, CA). HRR reporter pDR-GFP plasmid and transfection control pDsRed plasmid [32] were generously provided by Drs. EL Mustapha Bahassi and Peter Stambrook (University of Cincinnati, Cincinnati, OH). Lipofectamine 3000 and Alexa Flour secondary antibodies were obtained from ThermoFisher Scientific (Waltham, MA).

### Cells and culture

Prostate cancer cells (LNCaP, PC-3, and 22Rv1) and A549 lung cancer cells were obtained from ATCC (Manassas, VA). The cells were expanded and kept in cryogenic storage for long-term safekeeping. The cells were authenticated genetically with PCR identifying the short tandem repeat (STR) and cell-specific profiling against ATCC database at the University of Arizona Genetics Core. The cells were cultured in RPMI-1640 medium supplemented with 10% FBS at 37°C in 5% CO_2_. Cells in exponential growth phase were harvested by treatment for 1–3 minute with a 0.25% trypsin-0.02% EDTA solution and resuspended in the medium. Only suspensions of single cell with viability exceeding 95% (ascertained by trypan blue exclusion) were used in the study.

### Gamma- and ultraviolet-C (UV-C)-irradiation

For irradiation with a gamma-emitting cesium (Cs)-137 source, cells were detached, washed, resuspended in culture medium, and irradiated at desired doses in a Gamma Cell 40 Exactor research irradiator (Nordion, Ontario, Canada). For UV-C irradiation, cells were plated into 96-well plates in phenol red-free medium, allowed to attach overnight, and exposed to UV-C light from a germicidal lamp (G64T5L, Sankyo Denki, Japan) with an output of 25W at 253.7 nm and irradiance of 0.25 mW/cm^2^ at 1 m from the lamp in a tissue culture hood at the doses indicated in Results.

### MTT assay

Cells were plated into 96-well plates and treated with PCNA-I1S and/or other DNA damaging agents as indicated and cultured for 96 hours. At the end of the incubation, the cells in control and treated cultures were stained with MTT as described in our previous study [33]. Growth inhibition (%) in the treated cells relative to the untreated control cells was calculated using the formula: (1-*A*_570_ of treated cells/*A*_570_ of control cells) x 100, which reflects the effects of the treatments on cell growth.

### Immunofluorescent staining

Cells were seeded into chamber slides at 2 x 10^4^ cells/well and allowed to adhere overnight. The cells were then treated with PCNA-I1S and/or other DNA damaging agents for the times indicated. For chromatin-bound PCNA staining, PC-3 cells were treated with ice-cold buffer A [10 mM Tris-HCl, pH 7.4, 2.5 mM MgCl_2_, 0.5% Nonidet P-40 (NP-40), 1 mM dithiothreitol, 1 mM PMSF, and protease inhibitor cocktail] for 5 min, fixed with cold methanol, and rinsed with PBS containing 0.1% Tween-20. For γH2AX staining, A549 cells were fixed with 2% paraformaldehyde for 20 minutes, followed by washing twice with PBS containing 0.1% Tween-20 and permeabilized with methanol for 10 minutes at -20°C. The cells were then blocked using 5% normal goat serum for 1 hour at room temperature. Primary antibodies were diluted per the manufacturer’s recommendation and incubated overnight at 4°C. After washing, the cells were incubated with a fluorochrome conjugated secondary antibody for 1 hour at room temperature and mounted for analysis under an Olympus fluorescent microscopy. DAPI (4′,6-Diamidino-2-Phenylindole, dihydrochloride) was used as a nuclear counterstain to reveal all cells. Images were captured with a cooled CCD camera using Spot Advanced software (Spot Imaging Solutions, Sterling Heights, MI).

### Immunoblotting

Chromatin-associated PCNA was analyzed by immunoblotting as described in our previous studies [29, 30]. Cells were collected by trypsinization, pelleted (300 g, 5 min, 4°C), washed in PBS with 1 mM PMSF, and lysed in buffer A for 10 minutes. The samples were then pelleted by centrifugation (1,500 g, 5 min, 4°C) and the supernatant fraction collected as the NP-40-extractable (NP-E) fraction that contains free-form PCNA. The resulting pellet was washed in buffer B (10 mM Tris-HCl, pH 7.4, 150 mM NaCl, 1 mM PMSF, and protease inhibitor cocktail), resuspended and digested in buffer C (10 mM Tris-HCl, pH 7.4, 10 mM NaCl, 5 mM MgCl_2_, 0.2 mM PMSF, and protease inhibitor cocktail) with 200 units/10^7^ cells of DNase I for 30 min with agitation at room temperature. After centrifugation (13,000 g, 5 min, 4°C), the supernatant was collected as NP-40-resistant (NP-R) fraction that contains chromatin-bound PCNA. The two fractions of proteins were resolved by SDS-PAGE and analyzed by immunoblotting using PCNA antibody as well as antibodies to α-tubulin and histone H1 as loading controls for NP-E and NP-R proteins, respectively.

### The alkaline Comet assay

DNA damage in single cells was determined with a gel electrophoresis assay (the Comet assay). The alkaline Comet assay detecting DNA with both single-strand breaks (SSBs) and double-strand breaks (DSBs) [34, 35] was performed using the OxiSelect^™^ Comet assay kits following the manufacture’s instruction. After the staining with Vista Green DNA Dye (Cell Biolabs), the slides were examined under an Olympus fluorescent microscopy and images were captured. Images of the Comet assay from two experiments with 60–100 cells each treatment group were analyzed using OpenComet (v1.3.1), a plugin [36] for the imaging processing software Image J, to automatically calculate Tail DNA% (= 100 x Tail DNA Intensity/DNA Intensity) and Extent Tail Moment (= Length of Tail x Tail DNA%).

### Determination of HRR in a GFP reporter assay

The pDR-GFP HRR reporter is a mammalian expression vector containing a green fluorescent protein (GFP) expression cassette. pDR-GFP plasmid has 2 tandem but inactive GFP repeats, one of which contains the unique I-*SCE1* site. pDR-GFP was linearized by cleavage with *I-SCE1* endonuclease and purified. Upon transfection into cells, GFP expression is detected when a functional GFP protein is reconstituted by HRR-mediated homologous recombination between the 2 non-functional repeats [32]. PC-3 and A549 cells were plated into 96-well plates at 2 x 10^4^ cells/well in antibiotics-free media and allowed to adhere overnight. The cells were transfected with the linearized pDR-GFP HRR reporter or pDsRed (control vector) plasmids using Lipofectamine 3000 following the manufacturer’s instructions. Four hours later, PCNA-I1S was added to the cultures to treat the cells for 48 hours. Expression of GFP from pDR-GFP and red fluorescent protein (RFP) from control vector were examined and counted under an Olympus fluorescent microscopy, and images were captured.

### Assessment of UV-C-induced DNA damage

Cells in phenol red-free medium were plated into 96-well plates at 2 x 10^4^ cells/well and allowed to adhere overnight. The cells were exposed to UV-C, then cultured for 24 hours in the absence or presence of PCNA-I1S. The presence of cyclobutane pyrimidine dimers (CPD) induced by UV-C irradiation was immunofluorescently stained using the OxiSelect^™^ Cellular UV-Induced DNA Damage Staining Kits following the manufacture’s instruction and examined under an Olympus fluorescent microscopy and images were captured. DAPI (4′,6-Diamidino-2-Phenylindole, dihydrochloride) was used as a nuclear counterstain to reveal all cells. A reduction of CPD-positive cell population and/or CDP-staining intensity indicates the removal of CPD through NER in the cells.

### Statistical analysis

Data from each assay were expressed as means ± SD. Statistical differences between 2 groups were determined by the Student’s t test. Data from Comet assay was analyzed by one-way Analysis of Variance (ANOVA). All statistical analyses were carried out using Prism software (GraphPad, San Diego, CA). P < 0.05 was considered significantly different.

## Results

### The combinatorial effects of PCNA-I1S and Cs-137 irradiation on cell growth inhibition

**Fig 1:**
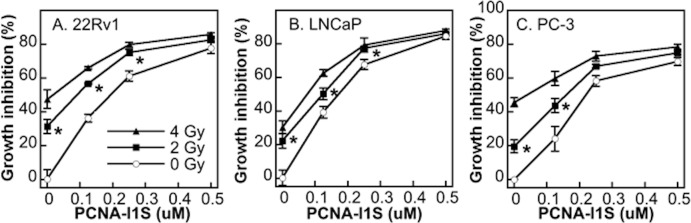
The combinatorial effects of PCNA-I1S and Cs-137 irradiation on cell growth inhibition. After irradiation with Cs-137 at 2 or 4 Gy, the cells were plated into 96-well plates (1,000 to 3,000 cells/well, 4 to 6 wells/treatment group) and allowed to attach for 3 hours. The cells were then treated with increasing concentrations of PCNA-I1S for 24 hours. After removing PCNA-I1S containing medium, the cells were then cultured in fresh medium for additional 72 hours and stained with MTT. Data shown are from one representative experiment of three. *, *p*<0.05 (Cs-137 irradiation alone vs. the combinations of Cs-137 irradiation and PCNA-I1S).

The measurement of *in vitro* tumor cell inhibition by PCNA-Is in MTT assay correlates closely with the measurement of cytotoxic activity of the compounds in colony formation assay and apoptosis assay [29, 31]. We assessed the combinatorial effects of PCNA-I1S with DNA damage agents on tumor cell growth using the MTT assay. First, we determined the combinatorial effects of PCNA-I1S with ionizing irradiation that mainly induces DNA SSBs and DSBs. PC-3, LNCaP, 22Rv1, and A549 cells were irradiated with Cs-137 at 2 or 4 Gy and plated into 96-well plates. Three hours later, the cells were treated with 0.125, 0.25, or 0.5 uM PCNA-I1S for 24 hours. The cells were then cultured in fresh medium for additional 72 hours in the absence of PCNA-I1S. As shown in Fig 1, the exposure to Cs-137 irradiation or PCNA-I1S alone led to the growth inhibition in a dose-dependent fashion in all cells. Moreover, the dose-dependent additive effects on cell growth inhibition were observed in cells exposed to combinations of Cs-137 irradiation and PCNA-I1S (Fig 1). Furthermore, we found that PCNA-I1S at 0.5 uM produced greatest growth inhibition either alone or in combination with Cs-137 irradiation.

### The combinatorial effects of PCNA-I1S and UV-C irradiation on cell growth inhibition

**Fig 2:**
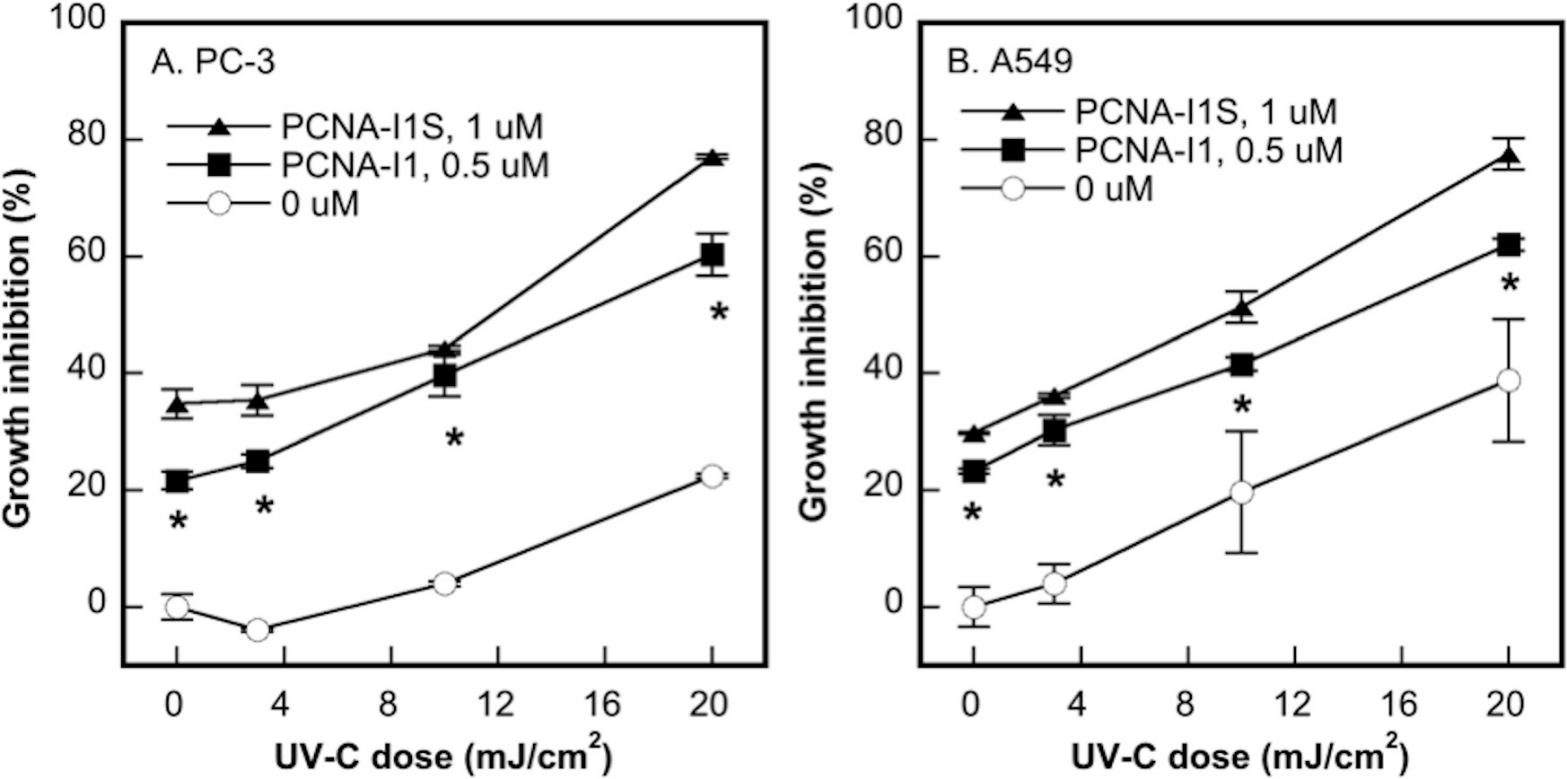
The combinatorial effects of UV-C irradiation and PCNA-I1S on cell growth inhibition. PC-3 (2,000 cells/well, 4 wells/treatment group) (A) and A549 (1,000 cells/well, 4 wells/treatment group) (B) in phenol red-free medium were plated into 96-well plates. After an overnight incubation, the cells were exposed to UV-C, followed by incubation for 24 hours in medium without or with either 0.5 or 1 uM PCNA-I1S. The cells were then cultured for 72 hours in fresh medium, followed by MTT assay. Data shown are from one representative experiment of three. *, *p*<0.05 (UV-C alone vs. the combinations of UV-C plus PCNA-I1S).

UV-C irradiation leads to the formation of three major types of DNA lesions, including CPDs, pyrimidine 6–4 pyrimidone photoproducts, and their Dewar isomers [37]. CDP in human cells is repaired mainly by NER [37, 38]. We determined the combinatorial effects of PCNA-I1S and UV-C irradiation on cell growth. PC-3 and A549 cells were irradiated with UV-C at 3, 10, or 20 mJ/cm^2^, followed by incubation in medium without or with 0.5 or 1 uM PCNA-I1S for 24 hours. The cells were then cultured for additional 72 hours in the absence of PCNA-I1S. As shown in Fig 2, UV-C irradiation induced growth inhibition in both PC-3 and A549 cells in a dose-dependent manner. Targeting PCNA with PCNA-I1S alone led to growth inhibition and produced additive effects on cell growth inhibition with UV-C irradiation (Fig 2).

### The combinatorial effects of PCNA-I1S and cisPt on cell growth inhibition

**Fig 3:**
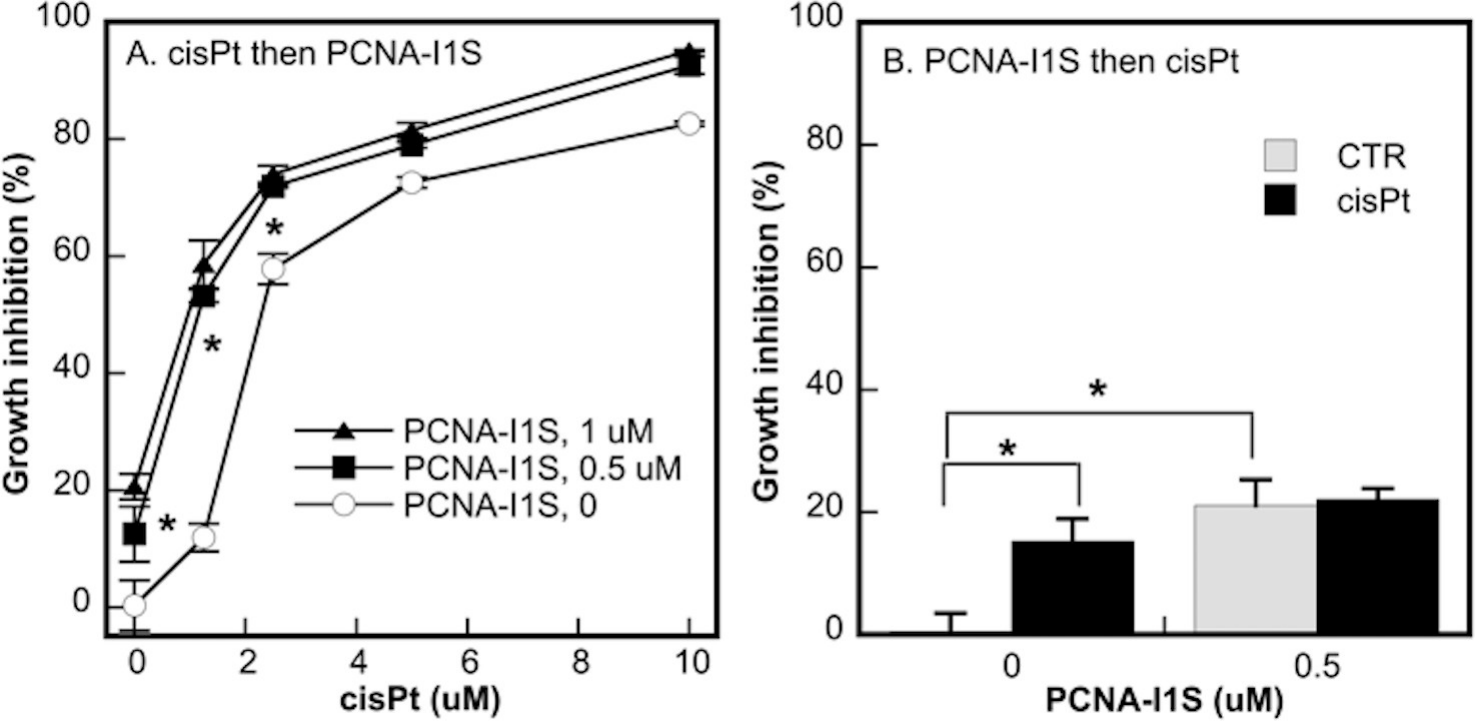
The combinatorial effects of cisPt and PCNA-I1S on cell growth inhibition. A549 cells (1,000 cells/well, 4 wells/treatment group) were plated into 96-well plates and incubated overnight. The cells were treated with cisPt (A) or PCNA-I1S (B) for 24 hours. After removal of cisPt or PCNA-I1S, the cells were treated with PCNA-I1S (A) or cisPt (B) for 24 hours. After an incubation for additional 48 hours in the absence of the drugs, cells in the cultures were subjected to MTT assay. Data shown are from one representative experiment of three. *, *p*<0.05 (Fig 3A, cisPt alone vs. the combinations of cisPt plus PCNA-I1S).

CisPt, a DNA damaging compound, is the first line chemotherapeutic agent used with another drug for treatment of advanced NSCLC [39]. We determined the combinatorial effects of PCNA-I1S with cisPt on growth inhibition in lung cancer cells. NSCLC A549 cells were treated with increasing concentrations of cisPt (1.5 to 12 uM) for 24 hours to induce DNA damages. After the removal of cisPt, the cells treated with PCNA-I1S (0.5 or 1 uM) for 24 hours. After incubation for additional 48 hours in the absence of the drugs, the cultures were stained with MTT. The treatment with cisPt or PCNA-I1S alone induced cell growth inhibition in a dose-dependent fashion (Fig 3A). Additive effects on cell growth inhibition were observed in cells exposed to combinations of cisPt and PCNA-I1S, most significantly at the low concentration of 1.5 uM cisPt (Fig 3A).

To explore the potential mechanisms underlying the additive effects of cisPt and PCNA-I1S on cell growth inhibition, A549 cells were treated with PCNA-I1S and cisPt in a reverse sequence as that in the experiment shown in Fig 3A, i.e., first with PCNA-I1S (0.5 uM) for 24 hours, followed by cisPt (1.5 uM) for 24 hours. The treatment with either PCNA-I1S or cisPt alone induced growth inhibition in A549 cells to the similar extent as those shown in Fig 3A (Fig 3B). However, the additive effects of PCNA-I1S and cisPt on cell growth inhibition were not observed in this reverse treatment setting (Fig 3B), suggesting that the additive effects are due to attenuation of DNA repair by PCNA-I1S.

### The effects of PCNA-I1S on PCNA association with chromatin and DNA damage

**Fig 4:**
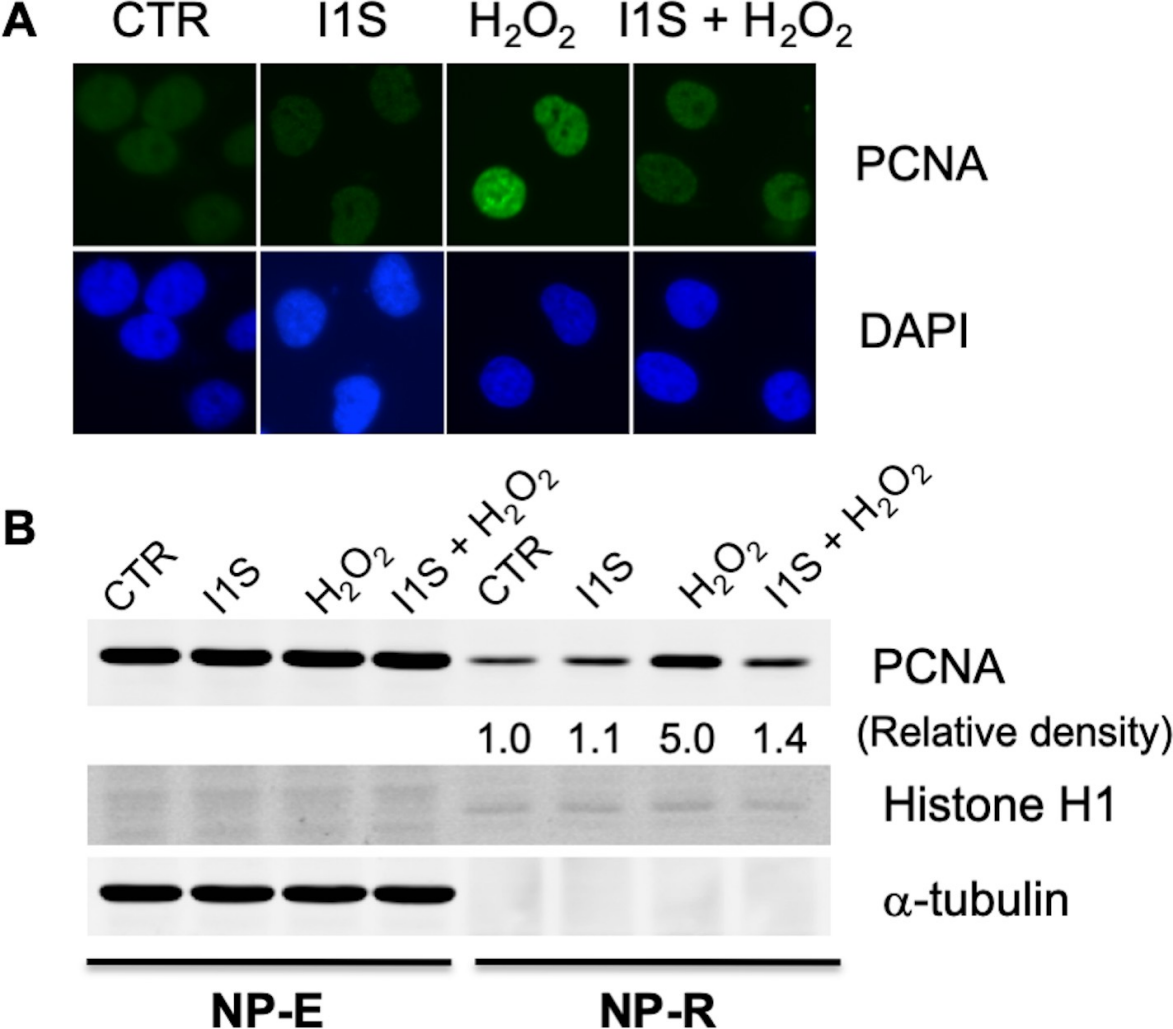
The effects of H_2_O_2_ and PCNA-I1S on PCNA association with chromatin. A. PC-3 cells (2 x 10^4^/well) were plated into chamber slides and incubated overnight. After starvation in serum-free medium for 3 days, the cells were treated with PCNA-I1S (1 uM) for 1.5 hours and/or H_2_O_2_ (100 uM) for 1 hour. The cells were lysed in a hypotonic buffer containing 0.5% NP-40, fixed in cold methanol, stained with PCNA antibody (PC10) and fluorescent secondary antibody. The cells were counterstained with DAPI to reveal cell density and nucleus. B. The cells were starved and treated as described in A. The NP-E and NP-R proteins were analyzed by immunoblotting to detect the free-form and chromatin-bound PCNA with α-tubulin and histone H1 levels as loading controls for NP-E and NP-R PCNA, respectively. The optical density of chromatin-bound PCNA was normalized to histone H1. The raw images of immunoblotting are provided as the supplementary data (S1 Raw Images).

PCNA is a nucleoplasmic protein and must be relocalized to execute its functions in response to various stimuli. In replicating cells, growth-stimulating signals induce PCNA relocalization to DNA replication forks to serve as platforms on DNA templates for partner proteins involved in DNA synthesis [2, 16, 40]. In quiescent cells, on the other hand, PCNA relocalizes to DNA repair foci upon DNA damage and is a critical component in multiple DNA repair pathways [41, 42]. Oxidative stress, spontaneous hydrolysis, and chemotherapeutic alkylating drugs cause DNA base damage, which is repaired by NER or BER, two processes requiring the participation of PCNA at DNA repair foci [42, 43]. PCNA relocalization to DNA repair foci induced by H_2_O_2_ is a reliable model for investigating oxidative stress-induced DNA damage and repair [42, 43]. We determined the effects of PCNA-I1S on oxidative stress-induced PCNA association to chromatin. To preferentially evaluate the association of PCNA with DNA repair foci, not the replication forks, we determined the effects of PCNA-I1S in PC-3 cells that were arrested at G1 phase of the cell cycle after starvation in serum-free medium for 72 hours [29]. The serum-starved PC-3 cells were incubated for 30 minutes in fresh medium or the medium with PCNA-I1S (1 uM) and then treated with H_2_O_2_ (100 uM) for 1 hour in the absence or presence of PCNA-I1S. After treatment with the hypotonic buffer containing NP-40 to release the free-form PCNA, chromatin-bound PCNA was revealed by immunofluorescent staining. As shown in Fig 4A, a low level of chromatin-bound PCNA was detected in the quiescent PC-3 cells. The chromatin-bound PCNA was greatly elevated in cells treated with H_2_O_2_ (Fig 4A). Treatment with PCNA-I1S significantly reduced H_2_O_2_-induced PCNA association with chromatin (Fig 4A). For validating the observation in immunocytochemistry, we performed immunoblotting. Data in Fig 4B shows that treatment with H_2_O_2_ and PCNA-I1S alone, or in combination did not significantly alter the level of free-form PCNA in the cells (NP-E fraction). In contrast, H_2_O_2_ enhanced the level of chromatin-bound PCNA (NP-R) by approximately 5 folds in the serum-starved PC-3 cells and the effect of H_2_O_2_ was significantly attenuated by PCNA-I1S.

**Fig 5:**
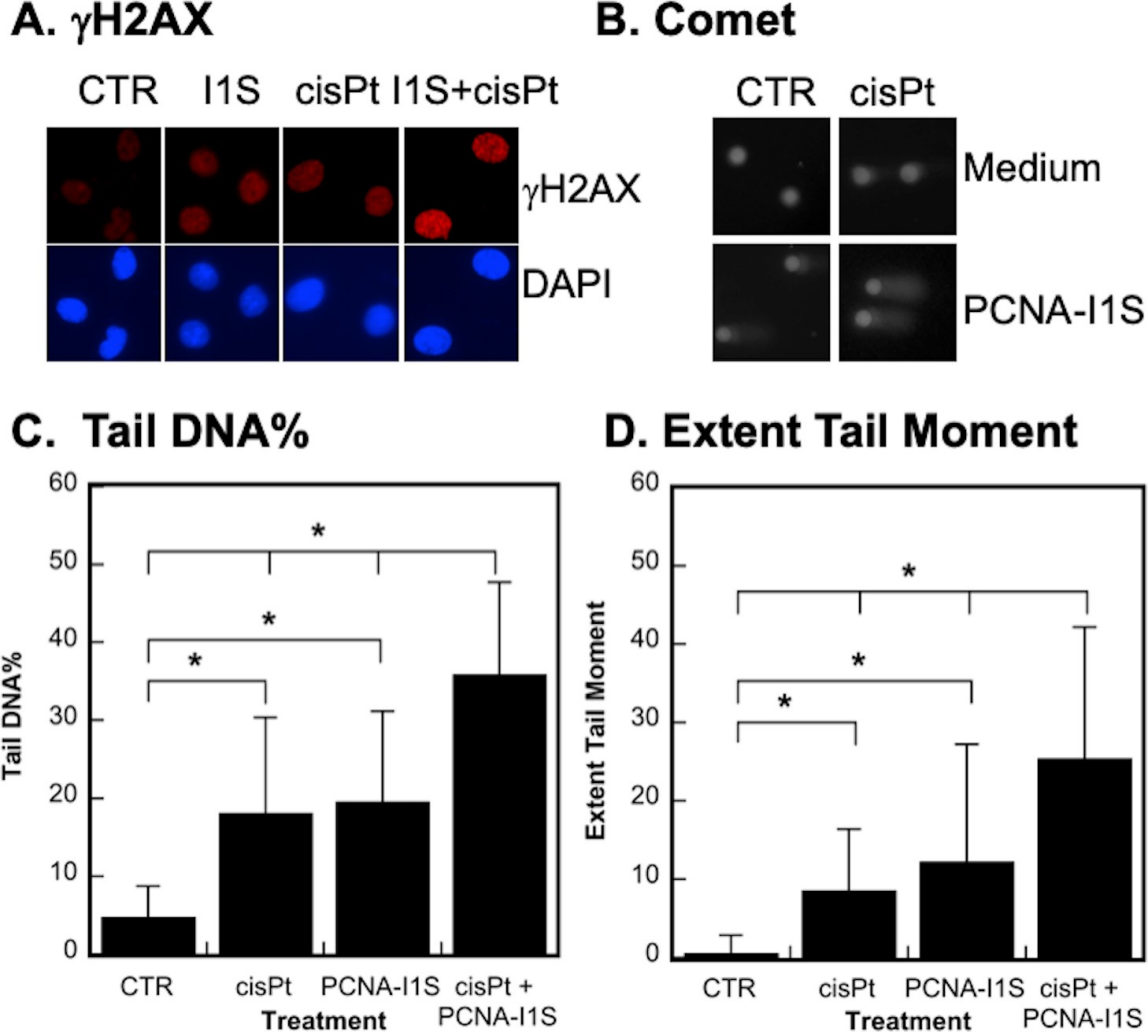
The effects of cisPt and PCNA-I1S on DNA damage. A. A549 cells (1 x 10^4^/well) were plated into chambered slides. After an overnight incubation. the cells were treated with PCNA-I1S and/or cisPt and immunofluorescently stained with antibody to γH2AX. The cells were counterstained with DAPI to reveal cell density and nucleus as controls. B. A549 cells (1 x 10^6^/plate) were plated into 60-mm plates and incubated overnight. After treatment with PCNA-I1S and/or cisPt, the cells were gently detached with a rubber scraper and analyzed with the Comet assay kits following the manufacture’s instruction. The images of Comet assay with 60–100 cells each treatment group were analyzed with OpenComet software to derive Tail DNA% and Extent Tail Moment, which were showed in C and D, respectively. *, *p* < 0.05 for all the comparisons as indicated in Fig 5C and Fig 5D.

CisPt binds to and induces DNA damages by forming monoadducts, intrastrand crosslinks, interstrand crosslinks (ICLs), and eventually DSBs [9, 44]. The accumulation of γH2AX is a reliable biomarker for DNA DSBs [45–47]. We determined the accumulation of γH2AX in A549 cells treated with PCNA-I1S and/or cisPt. As shown in Fig 5A, treatment with PCNA-I1S (1 uM) or cisPt (5 uM) alone for 24 hours increased γH2AX level in the cells. The expression of γH2AX was significantly elevated in cells treated with cisPt plus PCNA-I1S, suggesting an additive effect of PCNA-I1S and cisPt on DNA damage. We further determined the effects of cisPt and PCNA-I1S on DNA damage in single cells using the alkaline Comet assay that detects DNA SSBs and DSBs [34, 35]. Fig 5B shows that treatment with cisPt or PCNA-I1S alone induced DNA damage in A549 cells and the extent of DNA damage was further increased in the cells treated with cisPt plus PCNA-I1S. The images were quantitatively analyzed with OpenComet software to derive Tail DNA% and Extent Tail moment, two parameters recommended for monitoring the extent of DNA damage in mammalian cells [48]. Both Tail DNA% (Fig 5C) and Extent Tail Moment (Fig 5D) were significantly increased in cells treated with cisPt or PCNA-I1S alone. An additive effect of cisPt and PCNA-I1S was observed in the induction of Tail DNA% and Extent Tail Moment (Fig 5C and 5D).

### The effects of PCNA-I1S on DNA repair

PCNA is a critical component in multiple DNA repair pathways [49, 50], such as BER [42, 51], NER [50, 52], and HRR [50, 53]. The interference of DNA repair could be a major mechanism responsible for DNA damage (Fig 5) and growth inhibition (Figs 1–3) observed in PCNA-I1S-treated cells. We have shown that PCNA-I1S interferes with PCNA recruitment to chromatin induced by H_2_O_2_ (Fig 4). We further determined the effects of PCNA-I1S on DNA repair in two well-characterized model systems.

**Fig 6:**
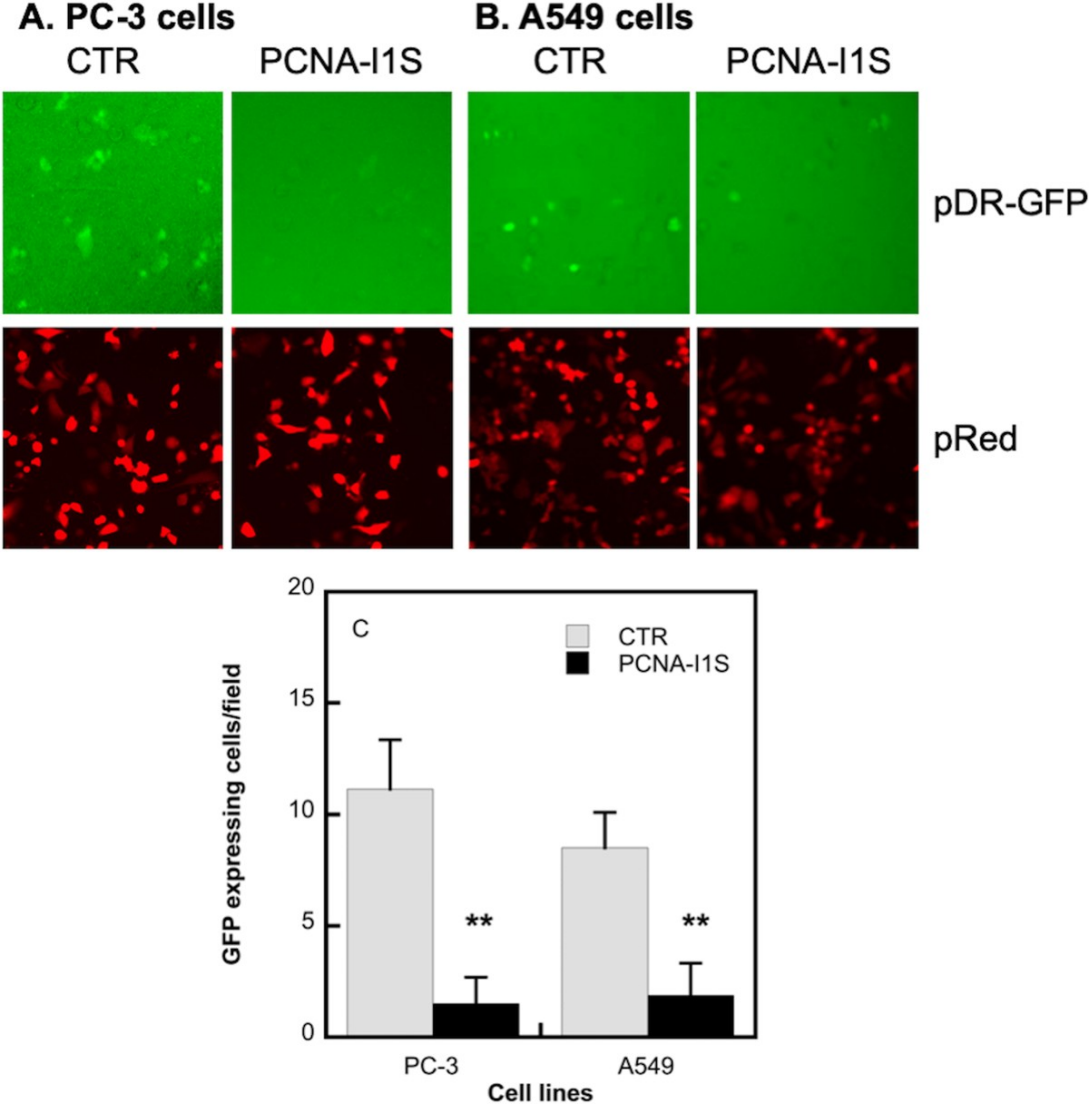
The effects of PCNA-I1S on HRR. PC-3 cells (2 x 10^4^cells/well, 6 well/treatment group) (A and C) and A549 cells (2 x 10^4^cells/well, 6 well/treatment group) (B and C) were plated into 96-well plates and incubated overnight. The cells were transfected for 4 hours with *I-SCE1-*linearized pDR-GFP or pDsRed using Lipofectamine 3000 and incubated for additional 48 hours in the absence or presence of 0.5 uM PCNA-I1S. GFP-expressing cells in 10 fields were counted and calculated (C). **, *p*<0.01.

DNA DSBs in cells are repaired mainly through HRR and the nonhomologous end joining (NHEJ) [50, 53, 54]. HRR requires the participation of PCNA [50, 53, 54], whereas NHEJ is largely PCNA-independent [55]. We determined the effects of PCNA-I1S on HRR, a pathway mainly responsible for repairing DSBs at the S and G2 phases of the cell cycle in replicating cells [56]. PC-3 and A549 cells were transiently transfected for 4 hours with linearized pDR-GFP or control plasmid pDsRed. The transfected cells were then treated for 48 hours with PCNA-I1S (0.5 uM). Expression of GFP, the indicator of HRR activity, in control and treated cells was examined. Data in Fig 6 showed that the numbers of GFP-expressing PC-3 cells (Fig 6A and 6C) and A549 cells (Fig 6B and 6C), but not those of pDsRed expressing cells, were significantly reduced by PCNA-I1S, indicating that PCNA-I1S attenuated HRR in the cells.

**Fig 7:**
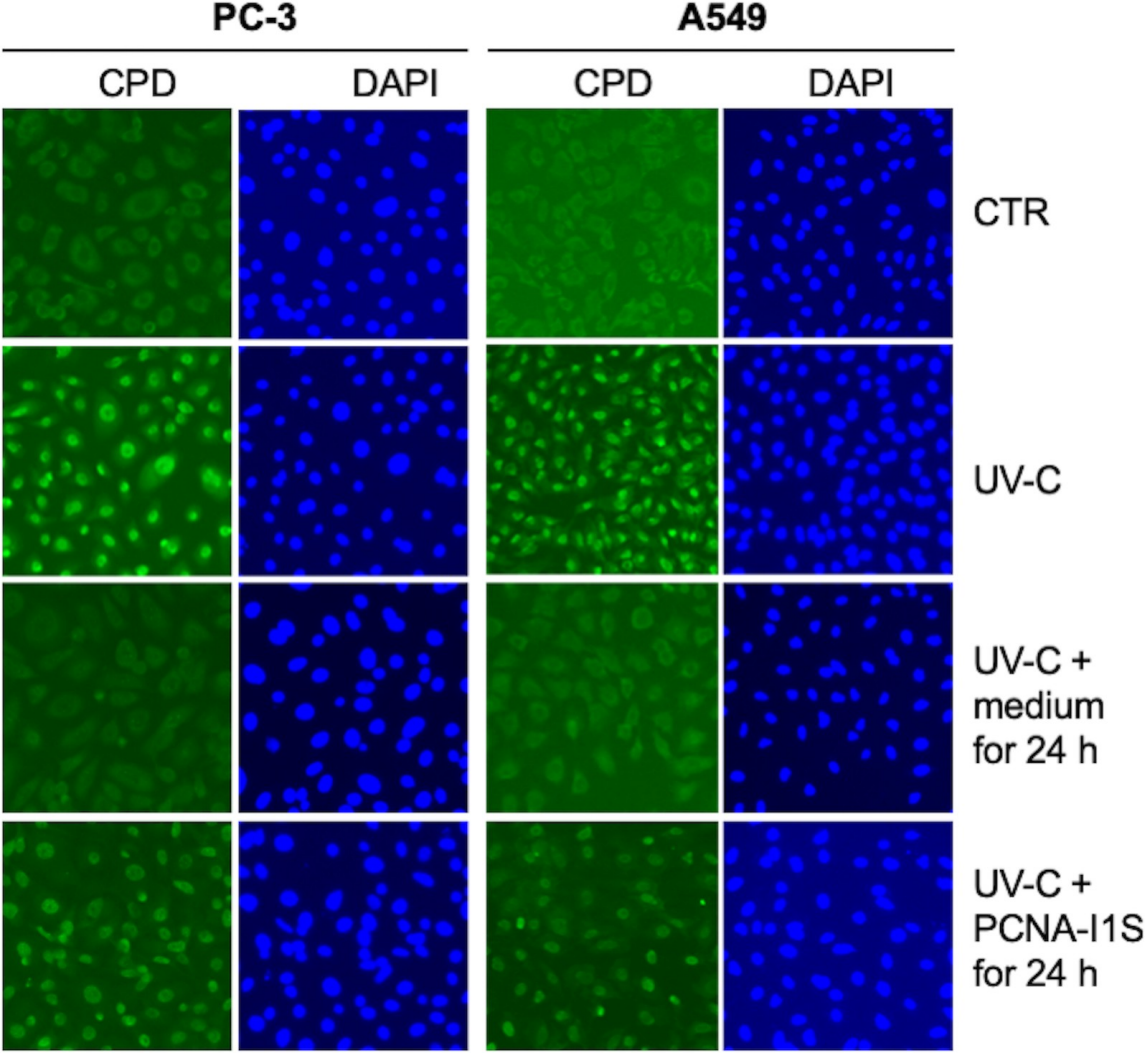
The effects of PCNA-I1S on repair of UV-C-induced CPD. PC-3 and A549 cells (2 x 10^4^cells/well) in phenol red-free medium were plated into 96-well plates and incubated overnight. The cells were exposed to 10 mJ/cm^2^ UV-, followed by incubation for 24 hours in the absence or presence of 0.5 uM PCNA-I1S. CPD in the cells were immunofluorescently stained in 30 minutes after UV-C irradiation or at end of the 24-hour incubation with kits following the manufacture’s instruction. The cells were counterstained with DAPI to reveal cell density and nucleus as a control.

UV irradiation induces two types of DNA damage: CPD and pyrimidine (6–4) pyrimidone photoproducts [50, 52]. CDP in cells is mainly repaired by NER. NER is also responsible for repair DNA damage induced by some chemotherapeutic agents, such as cisPt [57, 58]. Indeed, platinum drug resistance was shown to be associated with tumor cells with an elevated NER capacity [58]. We have observed the additive effects of PCNA-I1S and UV-C irradiation in the growth inhibition experiments (Fig 2). Using the UV-induced CPD as a model system [38], we further determined the effects of PCNA-I1S on repair of CPD. A strong nuclear CPD staining was detected in 30 minutes in all PC-3 and A549 cells upon exposure to UV-C at 10 mJ/cm^2^ (Fig 7, UV-C; CPD vs. DAPI), but not in the control cells (Fig 7, CTR). The nuclear CPD staining was not observed in the cells cultured in medium or treated with PCNA-I1S (0.5 uM) for 24 hours (S1 Supplementary Data). The number of CPD-positive cells, as well as CDP staining intensity, was significantly reduced in the cells exposed to 10 mJ/cm^2^ UV-C, followed by incubation for 24 hours in medium (Fig 7, UV-C + medium for 24 h), suggesting that CPD was repaired in the cells. In contrast, almost all cells (CPD vs. DAPI), that were exposed to UV-C, followed by incubation with PCNA-I1S (0.5 uM) for 24 hours, remained positive with nuclear CPD staining (Fig 7, UV-C+PCNA-I1S for 24 h). However, the intensity of nuclear CPD staining in these cells was significantly reduced in comparison with those stained in 30 minutes after UV-C irradiation (Fig 7, UV-C vs. UV-C+PCNA-I1S for 24 h). These data implicate that treatment with PCNA-I1S attenuated NER-mediated removal of CPD.

## Discussion

Previously we reported the identification and characterization of a novel class of small-molecule PCNA inhibitors that bind directly to PCNA trimers, stabilize the trimer structure, reduce PCNA association with chromatin, inhibit DNA replication, and selectively inhibit tumor cell growth [29]. Moreover, we found that treatment with the lead compound PCNA-I1 induces DNA damage and apoptosis, and suppresses tumor growth in mice [31]. Through the structure-activity relationship analysis and functional validation, we identified another lead compound PCNA-I1S that is more potent than PCNA-I1 in suppressing cell growth and stabilizing PCNA trimer structure [30]. The objective of the present study was to further investigate the effects of PCNA-I1S alone or in combination with several types of DNA damaging agents on cell growth inhibition, DNA damage, and to evaluate the effects of PCNA-I1S on DNA repair in prostate and lung cancer cells. Our data show that the combination of PCNA-I1S with DNA damage agents Cs-137 irradiation, UV-C irradiation, or cisPt produced significant additive effects on cell growth inhibition and DNA damage, and PCNA-I1S attenuated DNA repair in human prostate and lung cancer cells.

PCNA is indispensable for DNA replication and multiple DNA repair pathways [2, 11, 15, 16, 54]. Targeting PCNA with PCNA-I1S may inhibit cell growth through inhibition of DNA replication as well as attenuation of DNA repair. Suppression of PCNA relocalization to DNA replication forks will lead to stalling of DNA replication and increase susceptibility of DNA damage, resulting in the formation of DNA DSBs and cell growth inhibition [59]. Indeed, we found that targeting PCNA with PCNA-I1 inhibits DNA replication, induces S-G_2_-M phase arrest, results in the accumulation of DNA DSB marker γH2AX and activation of Chk2, increases expression of p53 and phosphorylation of p53, leading to growth inhibition [30, 31]. The similar effects of PCNA-I1 on cell growth and DNA damage were observed in cells treated with PCNA-I1S in the present study (Figs 1–4).

PCNA participates in multiple DNA repair pathways, such as BER, NER, HRR, and mismatch repair (MMR), which require DNA re-synthesis and involve PCNA interaction with DNA polymerase δ or polymerase ε [2, 16, 54]. In NER that repairs DNA damage induced by UV-C irradiation and some chemotherapeutic agents, such as cisPt [57, 58], PCNA facilitates the recruitment of the essential proteins endonuclease XP-G and XP-A for DNA repair [60, 61]. In BER that repairs DNA base damage induced by oxidative stress, spontaneous hydrolysis, and chemotherapeutic alkylating drugs [42, 43], PCNA interacts with polymerase β, and AP endonucleases Apn and Apn2, as well as other essential protein components [16]. For HRR that repairs DNA DSBs at S and G2 phases of the cell cycle in replicating cells [56], PCNA interacts with WRN helicase at the N-terminus domain [62]. Targeting PCNA with PCNA-I1S attenuated PCNA relocalization to chromatin (Fig 4), suppressed HRR- and NER-mediated DNA repair (Figs 6 and 7), and produced an additive growth inhibition with three types of DNA damaging agents (ionizing radiation with Cs-137, chemotherapeutic drug cisPt, and UV-C irradiation). Moreover, the combinatorial treatment of PCNA-I1S and cisPt produced additive effects on DNA damage (Fig 5). These observations confirmed and extended the findings reported by others on the additive effects of cisPt, bleomycin, or taxanes with small molecule PCNA inhibitors targeting PIP box [25, 63] or caPCNA [26], or a peptide inhibitor targeting APIM sequence [21, 28] on DNA damage, DNA repair, and cytotoxicity. Interestingly, we noted that the additive effects of PCNA-I1S with cisPt were observed only in the cells exposed to cisPt first, followed by PCNA-I1S, but not in those treated with the two agents in a reverse sequence. Given the significant inhibitory effects of PCNA-I1S on HRR- and NER-mediated DNA repair observed in this study, this data suggests that attenuation of DNA repair by PCNA-I1S is very likely the main mechanism responsible for the additive effects of PCNA-I1S with DNA damaging agents on tumor cell growth inhibition.

In conclusion, the data in this study provide strong evidence that targeting PCNA with PCNA-I1S suppresses PCNA association with chromatin in quiescent cells, inhibits DNA repair pathways, and produces the additive growth inhibitory effects with DNA damage agents. Moreover, the data suggest that targeting PCNA with these PCNA inhibitors may provide a novel approach for enhancing the efficacy of chemotherapy and radiation therapy in treatment of human prostate and lung cancers.

## Supporting information

S1 Raw Images Raw images of immunoblotting. (TIFF)

S1 Supplementary Data CPD was not detected in control (CTR) and PCNA-I1S-treated cells. PC-3 and A549 cells (2 x 10^4^cells/well) in phenol red-free medium were plated into 96-well plates and incubated overnight. The cells were incubated for additional 24 hours in the absence or presence of 0.5 uM PCNA-I1S. CPD in the cells were immunofluorescently stained with kits following the manufacture’s instruction. The cells were also counterstained with DAPI to reveal cell density and nucleus as a control.
(TIFF)
