# Supplementary figures and images for: Additive effects of a small molecular PCNA inhibitor PCNA-I1S and DNA damaging agents on growth inhibition and DNA damage in prostate and lung cancer cells
Source: PLoS One. 2019 Oct 10;14(10):e0223894. doi: 10.1371/journal.pone.0223894 (PMC6786632; doi:10.1371/journal.pone.0223894)

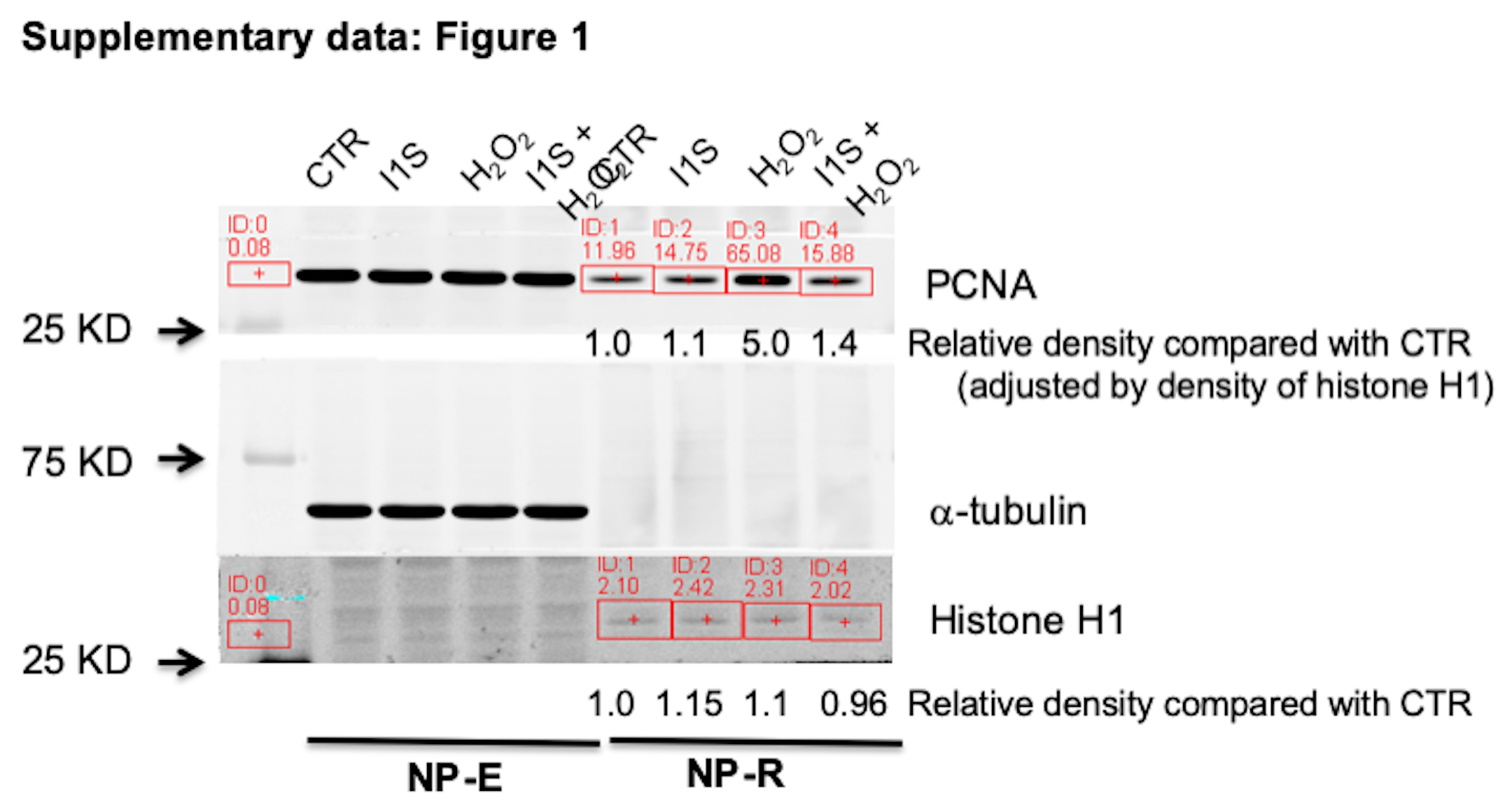

Supplement: S1 Raw Images — (TIFF) [file pone.0223894.s001.tiff]

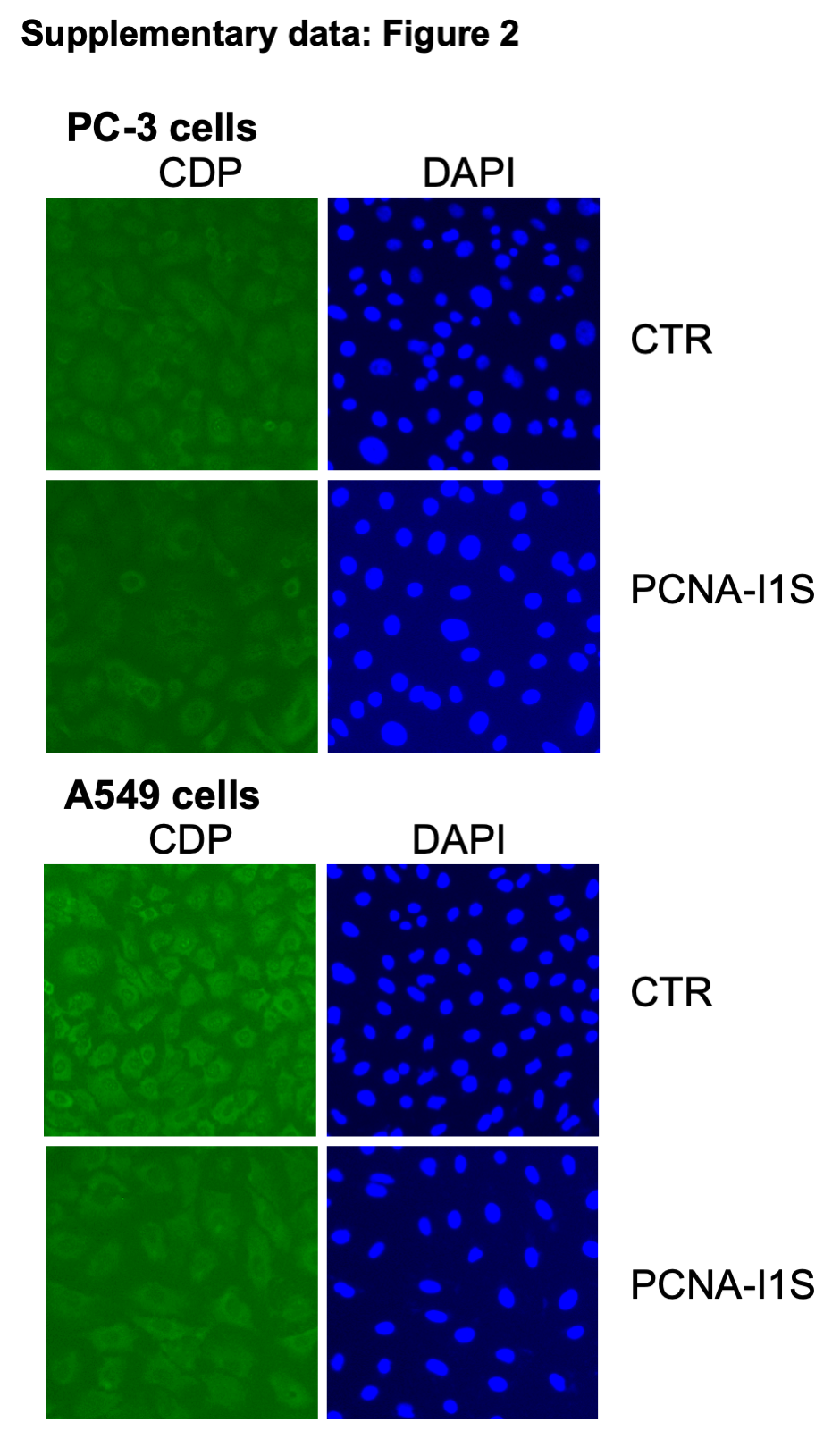

Supplement: S1 Supplementary Data — PC-3 and A549 cells (2 x 10^4^cells/well) in phenol red-free medium were plated into 96-well plates and incubated overnight. The cells were incubated for additional 24 hours in the absence or presence of 0.5 uM PCNA-I1S. CPD in the cells were immunofluorescently stained with kits following the manufacture’s instruction. The cells were also counterstained with DAPI to reveal cell density and nucleus as a control. (TIFF) [file pone.0223894.s002.tiff]
